# Overall Survival and Cancer-Specific Mortality in Patients with Prostate Cancer Undergoing Definitive Therapies: A Narrative Review

**DOI:** 10.3390/jcm13185561

**Published:** 2024-09-19

**Authors:** Makoto Kawase, Keita Nakane, Koji Iinuma, Kota Kawase, Tomoki Taniguchi, Masayuki Tomioka, Yuki Tobisawa, Takuya Koie

**Affiliations:** Department of Urology, Graduate School of Medicine, Gifu University, Gifu 5011194, Japan; nakane.keita.k2@f.gifu-u.ac.jp (K.N.); iinuma.koji.s0@f.gifu-u.ac.jp (K.I.); kawase.kota.b5@f.gifu-u.ac.jp (K.K.); taniguchi.tomoki.a8@f.gifu-u.ac.jp (T.T.); tomioka.masayuki.p7@f.gifu-u.ac.jp (M.T.); tobisawa.yuki.a7@f.gifu-u.ac.jp (Y.T.); koie.takuya.h2@f.gifu-u.ac.jp (T.K.)

**Keywords:** prostate cancer, overall survival, cancer-specific mortality, definitive therapy

## Abstract

The overall survival (OS) of patients with prostate cancer (PCa) who receive locally definitive therapy is generally better than that of patients who do not receive definitive therapy. There is no difference in the incidence of local recurrence or distant metastasis between treatment modalities. Because the prognosis of PCa is relatively good, many studies have focused on quality of life after treatment as an endpoint. However, a limited number of patients develop biochemical recurrence after definitive treatment for PCa and subsequently develop distant metastasis or die from PCa. Therefore, we believe that preventing local recurrence and distant metastasis and prolonging the OS should be emphasized when selecting a treatment modality for PCa. In this review, the significance and usefulness of radical prostatectomy and radiation therapy as the main modalities of definitive therapies for local PCa and locally advanced PCa were evaluated, as well as the outcomes of OS and PCa-specific mortality and the treatment options after biochemical recurrence to improve the oncological outcomes.

## 1. Introduction

Prostate cancer (PCa) is one of the most common malignant neoplasms affecting a large number of men [1]. In 2024, an estimated 299,010 men will be newly diagnosed with PCa, and an estimated 35,250 men will die of the disease, which is the second leading cause of cancer-related deaths [1]. In Japan, 94,748 patients were newly diagnosed with PCa in 2019 (the most diagnosed cancer in men), and 13,439 patients died due to PCa in 2022 (the seventh leading cause of cancer death) [2]. A large retrospective review of patients with PCa who underwent radical prostatectomy (RP) with a median follow-up of 5.3 years from 1982 to 1997 found a 15-year postoperative metastasis-free survival (MFS) rate of 82%, median time of 8 years from biochemical recurrence (BCR) to metastasis, and median time of 5 years from the development of metastatic disease to death [3]. The recent randomized controlled ProtecT trial examined the oncological outcomes in patients with PCa who underwent RP and radiation therapy (RT) and in those who chose monitoring surveillance (MS) [4]. Although the 10-year disease progression, metastatic, and PCa-specific mortality (PCSM) rates in RP were similar to those in RT, they were better than in MS [4]. Compared with MS for cancer-specific survival (CSS), MFS, and progression-free survival (PFS), the RP had hazard ratios (HR) of 0.32 (95% confidence interval [CI], 0.12–0.87; *p* = 0.026), 0.33 (95% CI, 0.19–0.55; *p* < 0.001), and 0.23 (95% CI, 0.15–0.33; *p* < 0.001), respectively, and the RT indicated HR of 0.34 (95% CI, 0.32–0.92), 0.40 (95% CI, 0.23–0.69; *p* = 0.001), 0.23 (95% CI, 0.17–0.32; *p* < 0.001), and 0.40 (95% CI, 0.23–0.69; *p* = 0.001), respectively [4]. However, there was no significant difference in the overall survival (OS) between treatment and MS, with HRs of 0.89 for RP and 0.95 for RT [4]. The OS of patients with PCa undergoing local definitive therapy is generally better than that of patients not undergoing local therapy, and several studies have reported no difference in the incidence of local recurrence or distant metastasis depending on the treatment method [5,6,7,8].

Conversely, several long-term follow-up studies have shown that 1.3–10% of patients die from PCa, although PCSM is lower with active surveillance (AS) or androgen deprivation therapy (ADT) [9,10,11]. Therefore, some patients develop BCR after definitive therapy for PCa, which subsequently leads to distant metastasis [12]. PCa that develops distant metastasis is associated with the transition from metastatic castration-sensitive PCa to metastatic castration-resistant PCa (mCRPC) and death from PCa at a median time of approximately 3 years, even if various therapies have been administered [13,14]. Many studies have focused on the quality of life (QOL) after treatment as an endpoint because PCa has a relatively good prognosis [15,16,17,18]; however, we believe that the importance of preventing metastasis and prolonging the OS should be emphasized when selecting a treatment for PCa. Therefore, the OS and PCSM should be considered as endpoints for the treatment of localized PCa (LPC) or locally advanced PCa (LAPC) [19].

In this review, the significance and usefulness of RP and RT as the main modalities of definitive therapies for LPC and LAPC were evaluated, as well as the outcomes regarding the OS and PCSM and the treatment options after BCR to improve the oncological outcomes.

## 2. Methods

**Figure 1:**
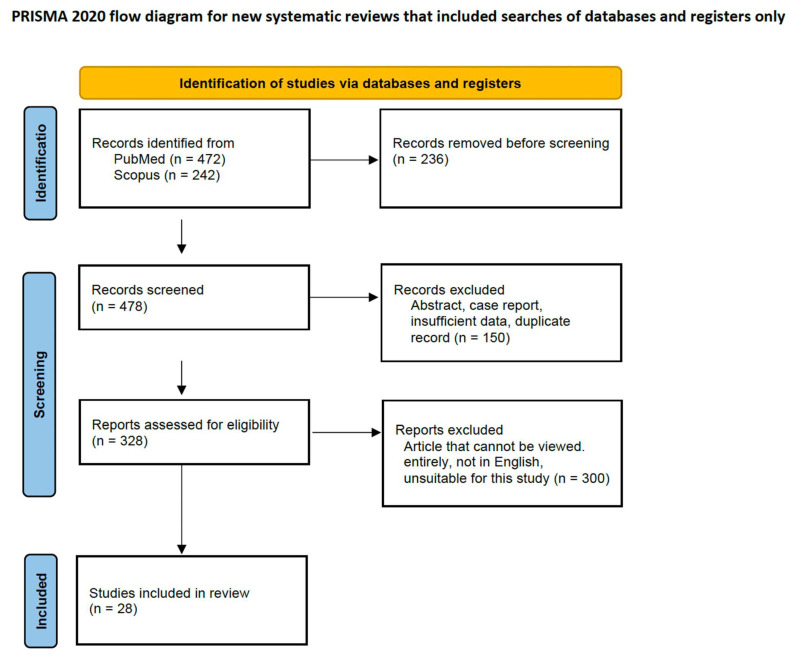
Study selection.

For this narrative review, the PubMed and Scopus online databases were searched for relevant studies. The eligible references included peer-reviewed English-language articles published from June 1993 to June 2024 containing the following medical subject heading terms: “prostate cancer”, “prostatectomy”, “radiation therapy”, “survival”, and “cancer-specific mortality”. The references of all the articles were scrutinized, and additional studies of interest were included. Meeting abstracts, case reports, studies with insufficient data, and duplicate records were excluded. Three authors (MK, KN, and KN) independently screened the literature, extracted the data, and assessed article quality. Discrepancies were resolved by consensus among the authors. A total of 328 references on definitive therapies for PCa, OS, and PCSM were found, of which 28 were selected based on the full-text availability in English (Figure 1).

## 3. Radical Prostatectomy

### 3.1. Surgical Outcomes of Robot-Assisted RP

RP and RT are currently the definitive treatment options for LPC or LAPC, according to several guidelines [20,21]. Among various RP surgical procedures, robot-assisted RP (RARP) is a minimally invasive procedure that offers several advantages in terms of blood loss, transfusion rate, postoperative pain, and a shorter hospital stay [22]. Over the past decade, RARP has become the most frequently performed surgical treatment for patients with LPC, LAPC, or metastatic PCa [23,24,25]. RARPs are increasingly performed using various instruments [26,27,28]. Therefore, trifecta and pentafecta criteria have been proposed as indicators of the quality of RARPs [29,30]. These concepts include the following five factors: undetectable prostate-specific antigen (PSA), urinary continence, potency, no operative complications, and negative surgical margin [29,30]. However, the operative time and estimated blood loss (EBL) would also be considered when assessing the actual RARP level. Therefore, the operation time, EBL, and surgical margins are discussed in this section.

Regarding the operative time, RARPs have previously tended to be significantly longer than open RPs (ORPs) [31,32]; however, the operative time is currently reported to plateau after operating on approximately 50–100 cases [27,33,34,35]. RARP has the potential to reduce intraoperative bleeding and blood transfusion rates because of its magnified field of view and more precise hemostasis, as well as because the surgery is performed after creating a pneumoperitoneum [36]. The results of a clinical study with a relatively large sample size comparing the surgical outcomes of ORP and RARP are listed in Table 1. Using data from the Mayo Clinic Prostatectomy Registry, 294 consecutive patients who underwent RARP for clinical LPC between August 2002 and December 2005 were compared in terms of surgical outcomes with those who underwent ORP at the same time [37]. In a study of approximately 200 initial cases compared to every 100 subsequent cases, the operative time for RARP was significantly prolonged compared to that for ORP (*p* < 0.001 and *p* = 0.004, respectively) [37]. For the latter 100 patients who underwent RARP, the median operative time was similar to that of ORP (211 vs. 228 min; *p* = 0.14) [37]. The perioperative blood transfusion rate in the ORP group was significantly higher than in the RARP group (*p* < 0.001) [36]. Rocco et al. [38] prospectively followed 120 patients undergoing RARP between November 2006 and December 2007 to evaluate the perioperative outcomes and compare them with a historical control group consisting of consecutive patients undergoing ORP. Although the overall mean operative time was significantly longer in the RARP than in the ORP group, “EBL was significantly lower in the RARP than in the ORP group (*p* < 0.001) [38]. Regarding the comparison of perioperative outcomes, although the operative time was significantly shorter in the ORP group than in the RARP group (*p* < 0.001), the intraoperative EBL was <500 mL in 98.4% of patients who underwent RARP compared to 69.7% of patients who underwent ORP (*p* < 0.001), indicating that RARP resulted in significantly less EBL [39]. Thus, RARP showed a statistically significant decrease in EBL and decreased need for blood transfusion compared with ORP in the majority of studies [36]. The need for a blood transfusion was significantly associated with the type of surgical procedure (RARP or ORP) and not with other parameters such as the patient’s age, body mass index, PSA level, or clinical stage in univariate or multivariate analyses [40].

In recent years, several reports have suggested that frailty and an age > 75 years do not affect the oncological outcomes of patients undergoing RARP, except for erectile function, which is adversely affected by advanced age [41,42,43]. Frailty has been associated with an increased incidence of perioperative complications and mortality 30 days after RARP, especially Clavien–Dindo grade >IV, as well as a higher incidence of moderate to severe postoperative pain [44,45]. A study of perioperative and oncological outcomes at a single institution in 630 consecutive patients with PCa who underwent RARP and were divided into two groups according to age (≥75 and <75 years) found no significant differences in the oncologic outcomes between the two groups [42]. Although the length of hospital stay tended to be longer in the older group (*p* = 0.051), the rate of Clavien ≥3 complications occurring within 1 month after RARP was similar in the two groups (*p* = 0.359) [42]. Therefore, careful evaluation of frail and older patients is of utmost importance with respect to their ability to tolerate surgery, and the use of accurate and easy-to-use geriatric screening tools may improve the outcomes by selecting patients who should undergo RARP [41].

Recent reports on operative time have reported a median operative time of 100–160 min for RARP [23,34,35]. RARP is expected to become a mainstream surgical procedure for patients with various complications, in addition to LPC and LAPC, and its indications will continue to expand. In addition, sequential surgical education of young physicians regarding RARP will enable them to master the technique of RARP with a short learning curve [35].

### 3.2. BCR after RARP

The ultimate goal of curative RP is to prevent clinical progression and death due to PCa. However, many patients, especially those with LAPC or high-/very-high-risk PCa, according to the National Comprehensive Cancer Network (NCCN) guidelines, have developed BCR after RARP [21]. BCR after RARP is generally defined as the time at which the postoperative serum PSA level rises to 0.2 ng/mL and persists after four weeks [46]. If the PSA level does not decrease to 0.2 ng/mL after RARP, the date of surgery is defined as the date of BCR as a persistent disease.

In a retrospective study of 374 patients with PCa who underwent RARP, 40 (10.7%) showed BCR during the follow-up period, at a median of 51 months [47]. In a multivariate analysis, the pathological stage ≥T3, pathological Gleason score (GS) ≥8, and a positive resection margin (RM1) were significantly associated with BCR [47]. With respect to BCR, a pathological T stage was associated with a 6-fold increased risk (odds ratio [OR], 6.31; 95% CI, 1.20–33.4; *p* = 0.030), a pathological GS with a 2-fold increased risk (OR, 2.10; 95% CI, 1.05–4.19; *p* = 0.036), and a RM1 with a 3-fold increased risk (OR, 3.02; 95% CI, 1.44–6.32, *p* = 0.003) [47]. In a single-center prospective cohort study of 885 patients with PCa undergoing only RARP, 167 (17.6%) had a median time to BCR of 2.9 years (interquartile range [IQR], 1.3–5.9 years), and the BCR-free survival (BRFS) was 81.8% (95% CI, 79.1–84.2%) at a median follow-up of 10.5 years [48]. In the multivariate analysis, the pathological stages T3a (HR, 1.6; 95% CI, 1.1–2.3; *p* = 0.01) and T3b (HR, 3.9; 95% CI, 2.2–6.8; *p* < 0.001) and a RM1 length >3 mm or multifocal (HR, 2.9; 95% CI, 1.9–4.5; *p* < 0.001) were significant predictors of BCR [48]. In contrast, no statistical difference was found with respect to BCR when the RM1 length was ≤3 mm (*p* = 0.24) [48]. At a median follow-up of 121 months (IQR, 97–132 months) of 483 patients with LPC who underwent RARP, 108 (22.4%) had BCR [49]. The 10-year BRFS was 73.1% (95% CI, 68.3–77.8), and at least 30% of patients diagnosed with low- or intermediate-risk PCa by the D’Amico risk classification developed BCR more than 5 years after RARP [49]. In the multivariate analysis, the pathological GS and RM1 were independent predictors of BCR (both *p* < 0.001) [49].

Regarding the surgical outcome of RP, PSM was reported in 6.5–32% of patients with PCa, showing a similar incidence of PSM after RARP, retropubic RP, and laparoscopic RP [50]. PSM after RP has been suggested to be associated with clinical progression and a decreased CSS [51]. Although the impact of PSM after RP on oncologic outcomes remains controversial, it has been reported that 27–44% of patients experienced BCR, 6.8–24% showed systemic progression, and 0.8–3.7% showed PCa-related death during 7–13 years of follow-up [52,53]. Table 2 presents the PSM rates, locations, and oncological outcomes of patients with PCa who underwent RARP [54,55,56,57,58]. Of the 872 consecutive patients with PCa who underwent RARP, 120 (14.7%) showed BCR during a median follow-up period of 27.8 months [54]. Patients with a PSM had a significantly higher incidence of BCR than those without PSM (*p* < 0.001) [54]. Apical and other PSMs (HR, 3.61; 95% CI, 1.70–7.65; *p* < 0.001), apical-only PSM (HR, 2.32; 95% CI, 1.30–4.12; *p* = 0.004), and non-apical PSM (HR, 4.19; 95% CI, 2.56–6.89; *p* < 0.001) were correlated with an increased risk of BCR compared with those without a PSM [54]. In the multivariate analysis, apical PSM alone (HR, 2.32; 95% CI, 0.97–3.16; *p* = 0.059) was not an independent predictor of BCR, whereas apical and other PSMs (HR, 2.22; 95% CI, 1.01–4.86; *p* = 0.044) and non-apical PSM (HR, 2.54; 95% CI, 1.54–4.18; *p* < 0.001) were associated with an increased risk of BCR [54]. Among patients with PSM, 52.7% had at least two PSMs, and 47.3% had a solitary PSM [55]. Of the patients with isolated PSM, 54.3% had apical PSM, 20.0% had mid-prostate PSM, and 25.7% had basal PSM [55]. According to the multivariate regression analysis, lymph node involvement (HR, 11.948; 95% CI, 3.803–37.533; *p* < 0.001), a GG ≥2 in PSM (HR, 3.281; 95% CI, 1.190–9.045; *p* = 0.022), and a maximum PSM length >6 mm (HR, 4.194; 95% CI, 1.620–10.858; *p* = 0.003) were significant predictors of BCR [55]. Although PSM has been found to be a useful predictor of BCR, some studies have suggested that the true usefulness of PSM as a predictor may be influenced by the location and number of PSMs as well as their length.

## 4. Radiation Therapy

### 4.1. Therapeutic Characteristics of RT for PCa

RT is a commonly used treatment option for LPC, LAPC, and metastatic PCa [59,60]. As an alternative to RP, especially in patients with LPC or selected LAPC without metastases, definitive external-beam radiation therapy (EBRT) and/or brachytherapy (BT), which treat the prostate and seminal vesicles without injury, may be used and have an effectiveness comparable to RP in all NCCN risk groups [21,59]. In addition, the role of RT continues to expand to treat patients with LAPC, de novo metastatic disease, and oligoprogressive disease [60]. Currently, the common techniques for RT of the prostate in patients with PCa include EBRT with conventional, moderate, and ultra-hypofractionation; low- or high-dose-rate BT; charged-particle therapy; or a combination of these methods, depending on the risk of PCa [60]. The radiotherapy modalities for PCa, according to the risk categories based on various guidelines, are listed in Table 3 [20,21].

The determination of the dose and fractionation of EBRT for treating malignant neoplasms is generally dependent on the sensitivity of tumor cells and adjacent normal tissues [60]. Conventional RT (C-RT) for LPC consists of daily 1.8- to 2-Gy fractions over 8 weeks [60]. Several prospective trials have established that doses of >78 Gy are associated with improved BRFS [61,62,63]. EBRT, with moderate hypofractionation, has the advantage of fewer fractions at 4–6 weeks compared to conventional fractionation, although the radiation dose per fractionation is usually higher (2.5–3 Gy) [60]. A prospective phase III dataset proved that moderate hypofractionation is not inferior to conventional fractionation in terms of BRFS in low- and intermediate-risk patients [64,65]. In addition, several studies on its safety and efficacy in patients with high-risk PCa have reported that irradiation modalities with moderate hypofractionation show antitumor efficacy comparable to conventional fractionation [66,67]. Therefore, a moderate hypofractionation regimen is recommended as one of the standard treatments with EBRT for PCa in the NCCN and American Society for Radiation Oncology/American Urological Association guidelines [20,21]. In contrast, charged-particle therapy, including proton beams, for PCa has attracted interest because of its biological and dosimetric advantages over C-RT [60]. The physical properties of charged particles include small and constant energy loss, rapid energy accumulation proximal to the target organ, and a steep energy drop distal to the tumor, with little or no dose effect on the surrounding organs other than the target organ [68]. These physical properties are expected to reduce the integral dose associated with photon therapy and minimize the amount of non-tumor-surrounding tissue exposed to low doses of radiation [68]. However, the use of charged-particle therapy over C-RT for the treatment of PCa is currently controversial. Although various guidelines recommend charged-particle therapy as the definitive therapy for PCa [20,21], several studies are ongoing to verify the true benefits of charged-particle therapy for PCa [68,69].

BT is a modality of RT in which a sealed radioisotope is implanted in the prostate to deliver a high dose of radiation to the target organ over a short distance while minimizing the dose to adjacent normal tissues [70]. BT has been the treatment of choice as monotherapy in low- and favorable-intermediate-risk PCa, and in combination with EBRT in unfavorable-intermediate- and high-risk PCa as the definitive treatment, with a low dose rate (LDR) or high dose rate (HDR) [15,16,70,71,72,73,74,75,76,77]. Additionally, the selection criteria for performing BT should consider not only the clinical factors such as prostate size, PCa grade, and extent of locally advanced progression but also age and preoperative urinary status [15,71,72]. In LDR-BT, a permanent radioactive seed implant with 125-iodine is placed in the prostate during a single surgery [15,71,72]. Seed insertion is performed using transrectal ultrasonography, and PCa is treated with a radiation dose of 0.4 to 2.0 Gy per hour from a radioisotope needle implanted in the prostate [74]. A randomized controlled RTOG 0232 trial showed that the combination of LDR-BT and EBRT was associated with increased late gastrointestinal and genitourinary toxicity compared with LDR-BT alone, although the oncologic outcomes were comparable, suggesting that LDR-BT alone should be recommended as a treatment modality for mainly favorable-intermediate-risk patients with PCa [75]. Regarding the benefit of combined EBRT and LDR-BT in intermediate- and high-risk patients with PCa, the phase III randomized Androgen Suppression Combined with Elective Nodal and Dose Escalated Radiation (ASCENDE-RT) trial compared the therapeutic outcomes of dose-escalating EBRT alone with EBRT that was accompanied by dose escalation with LDR-BT boosted to the prostate [76,77]. This study showed that the addition of LDR-BT to EBRT improved BCR by nearly 20% at 10 years, with no difference in the distant metastasis rates or PCSM [76,77]. In HDR-BT, a catheter needle is used to temporarily implant a highly radioactive source, 192-iridium, into the prostate under ultrasound guidance, and the catheter needle is removed after one to several doses of prescribed radiation [74,78]. Compared to LDR-BT, HDR-BT is a treatment modality that delivers radiation at a rate of ≥12 Gy/hour [78]. In a randomized phase III trial by Hoskin et al. [79], patients with intermediate- or high-risk LPC were randomized to receive hypofractionated EBRT (H-RT) alone or EBRT with a HDR-BT boost (HDR boost = 2 × 8.5 Gy delivered over 24 h) [79]. The 12-year follow-up data showed that the combination of EBRT and a HDR-BT boost significantly improved BRFS and was comparable to the EBRT monotherapy in terms of severe late uremia and enterotoxicity, making it a recommended monotherapy for low- and intermediate-risk PCa and a combination therapy for high-risk PCa [79]. Although LDR- and HDR-BT have shown nearly equivalent oncological outcomes, HDR-BT, which offers more flexibility in directing the dose outside the prostate for LAPC, appears to be the treatment of choice [60]. Regarding QOL after BT, 195 patients with PCa treated with BT were randomized: 108 to HDR and 87 to LDR [80]. The Baseline Expanded Prostate Cancer Index Composite (EPIC) scores were comparable between the LDR and HDR cohorts, with scores of 89 and 88 for the urogenital and 92 and 93 for the gastrointestinal categories, respectively [80]. Regarding the EPIC urologic score, the HDR group declined at 1 month but quickly stabilized at 6 months, whereas the LDR score was at its lowest at 3 months and then took 18 months to recover, after which urinary QOL remained similar in the HDR and LDR groups [80]. Bowel QOL scores declined in both cohorts, reaching their lowest values at 12 months, respectively [80]. Although the HDR group recovered to near baseline and maintained higher scores than the LDR group for up to 5 years, the LDR group scores remained significantly lower than the HDR group scores at 5 years [80]. Urinary QOL improved over time in both groups and was similar 18 months postoperatively; however, LDR tended to result in persistent bowel symptoms [80].

### 4.2. BCR after RT

The randomized phase III non-inferiority NRG Oncology RTOG 0415 trial compared conventional fractionated (73.8 Gy in 41 fractions) radiation therapy (C-RT) to hypofractionated (H-RT; 70 Gy in 28 fractions) in patients with low-risk PCa [81]. This trial included 1092 eligible patients and reported the results after a median follow-up of 12.8 years [81]. The estimated 12-year disease-free survival (DFS) was 56.1% for C-RT and 61.8% for H-RT, with a HR for DFS of 0.85 (*p* < 0.001) [81]. Long-term DFS was non-inferior in H-RT compared with C-RT [81]. A meta-analysis of published randomized clinical trials comparing moderate H-RT with C-RT was conducted to determine the efficacy of moderate (2.5 to 4 Gy) H-RT for LPC [82]. A total of 7 of the 365 eligible studies met the inclusion criteria, with 8156 patients with PCa enrolled [82]. Compared to C-RT, moderate H-RT had lower BCR rates (HR, 0.80; 95% CI, 0.68–0.95; *p* = 0.009), whereas the OS was similar in both groups (HR, 0.68; 95% CI, 0.78–1.02; *p* = 0.10) [82]. Although the biochemical and clinical disease failure (BCDF) rates were not significantly different between H-RT and C-RT (HR, 0.92; 95% CI, 0.82–1.02; *p* = 0.12), dose-escalation H-RT significantly reduced the BCDF rates compared to C-RT (HR, 0.84; 95% CI, 0.73–0.96; *p* = 0.01) [82]. This meta-analysis concluded that moderate H-RT did not improve the OS but reduced the BCR rates [82]. Moreover, the PACE-B trial demonstrated that H-RT (36.25 Gy in 5 fractions) was not inferior to C-RT for the 5-year BCDF rates in patients with clinical T stages 1 and 2, a GS ≤ 3 + 4, and a PSA ≤ 20 ng/mL [83].

BCR was studied in 476 patients with PCa who underwent LDR-BT, including 369 patients who underwent neoadjuvant ADT [84]. At a median observation period of 84 months, 20 patients (4.2%) showed BCR, with 3-, 5-, and 10-year BRFS rates of 97.9%, 96.9%, and 94.8%, respectively [84]. In a study of 993 patients diagnosed with intermediate-risk PCa, 775 were treated with LDT-BT alone and 158 with LDR-BT plus EBRT [85]. Propensity score matching was performed, and 102 pairs were investigated and followed for 95 months [85]. The 8-year BRFS was 93.3% for patients who received LDR-BT alone and 88.4% for those who received LDR-BT plus EBRT, with the former group performing significantly better (HR, 0.40; 95% CI, 0.158–0.911; *p* = 0.047) [84]. Of the 141 consecutive patients diagnosed with LPC who underwent HDR-BT alone, 23 (16.3%) showed BCR at a median follow-up period of 15.2 years, with 15- and 18-year BRFS rates of 85.1% and 78.7%, respectively [86]. Similarly, in 149 patients with intermediate-risk PCa treated with HDR-BT alone, the 5- and 8-year BRFS rates were 97% and 90%, respectively, with a median follow-up period of 6.2 years [87]. BCR was observed in 1293 patients with PCa, including 697 low-risk and 596 intermediate-risk patients who underwent HDR-BT or LDR-BT monotherapy, C-RT, or moderate H-RT [88]. No significant differences were found between the low- and intermediate-risk patients with respect to BCR (*p* = 0.31 and 0.72, respectively) [88]. The 5-year BRFS rates were 93–95% for low-risk PCa and 88–94% for intermediate-risk PCa, with no significant difference in BCR between the treatment modalities [88].

## 5. Oncological Outcomes

### 5.1. Oncological Outcomes after RP or RT

The oncological outcomes of patients with PCa who underwent definitive therapies for the prostate are listed in Table 4 [9,89,90,91,92,93]. Despite differences in patient backgrounds in each study, patients with PCa who underwent RP tended to have a prolonged PCSM and OS compared to those who underwent RT [9,87,88,89,90,91]. At present, however, data comparing the oncological outcomes of patients with PCa undergoing RP and RT should be interpreted with caution [84]. This is partly due to the different definitions of BCR for each treatment modality, which makes it fundamentally difficult to directly compare the oncologic outcomes of RT and RP [5,46].

A serum PSA level of >0.2 ng/mL is the most common definition of BCR after RP, although Phoenix’s definition of 2 ng/mL from the nadir point after treatment is commonly used for patients receiving RT, with a difference of approximately 10-fold between these two definitions [5,46]. The widely adopted Phoenix definition of BCR after RT is based on the rationale that EBRT generally preserves PSA-secreting glands, thus avoiding a large number of false positives [94]. However, if the two definitions for patients with PCa undergoing RP or RT are assessed fairly, the RT definition seems to have a greater probability of resulting in a large lead-time bias in favor of RT in the reporting of actuarial results [89]. When the RP definition was replaced with the Phoenix definition for all enrolled patients from the ASCENDE-RT database, the number of BCRs doubled, and the crude recurrence rate increased from 18% to 36% [94]. The 5-, 7-, and 9-year BRFS rates were 66%, 51%, and 57%, respectively, for the RP definition, compared with 87%, 82%, and 73%, respectively, for the Phoenix definition [94]. Although the Phoenix definition certainly seems useful for determining recurrence after RT, it may not be appropriate currently, considering that it has been more than 15 years since this definition was proposed and that RT has a variety of treatment options [84,95]. The definition of recurrence after RT may also need to be reevaluated in the future, given that metastasis and death due to PCa occur in >10% of patients who undergo RT [96]. In a meta-analysis comparing the efficacy of RP and RT for LPC, the 5- and 10-year CSSs were significantly longer for RP, with ORs and 95% CIs of 1.96 (1.42–2.72) and 2.44 (1.33–4.48) [97]. However, the HR for CSS in patients with low- to intermediate-risk PCa was not significantly different when examined by risk stratification [97]. A meta-analysis by Wallis et al. [98] reported that the OM and PCSM rates were higher in patients treated with RT than in those treated with RP. A subgroup analysis by risk group, radiation regimen, duration, and follow-up also showed that RP might contribute to an improved mortality compared to RT [98]. Compared to MS, RP and RT had higher rates of treatment-related adverse events: 95% had sexual dysfunction at 6 months postoperatively, 95% had urinary incontinence at 6 months postoperatively, and 20% at 3 years postoperatively for RP; 88% had sexual dysfunction at 6 months postoperatively, 65% had nocturia at 6 months postoperatively, and 5% had gastrointestinal dysfunction at 6 months postoperatively (for RT) were observed [4].

### 5.2. Treatment Method and Its Efficacy for Patients with mCRPC after RP and RT for the Prostate

The combination of abiraterone acetate and prednisone improved the OS and radiation PFS for chemotherapy-naïve mCRPC in the COU-AA-302 trial [99]. A total of 1053 patients were eligible for the secondary analysis reported in 2023 [100]. Of all patients, 64% (*n* = 669) received definitive therapy as prior therapy, of whom 53% (*n* = 353) received RT alone, 16% (*n* = 105) received RP alone, and 31% (*n* = 211) received a combination of RP and RT [100]. Additionally, significant reductions in the risk of death were associated with RP (HR, 0.51; 95% CI, 0.37–0.71), RT (HR, 0.80; 95% CI, 0.64–1.00), and RP plus RT (HR, 0.65; 95% CI, 0.50–0.85) [99]. Although the TAX327 and TROPIC trials demonstrated the efficacy of definitive therapy in mCRPC, no subgroup analysis has been performed [101,102]. Therefore, the efficacy of definitive therapy for patients with mCRPC receiving chemotherapy remains unclear. Of the 275 patients with mCRPC who received radium-223, 132 received definitive therapy (48%), of whom 93 received RP and 76 received RT [103]. Patients who received definitive therapy had a significantly longer survival than those who did not, with an estimated median survival of 18 and 11 months, respectively (*p* < 0.001) [103]. In addition, a report on the efficacy of radium-223 for mCRPC after RP showed that patients who underwent RP had a significantly lower mortality rate than those who did not (*p* = 0.04) [104].

**Figure 2:**
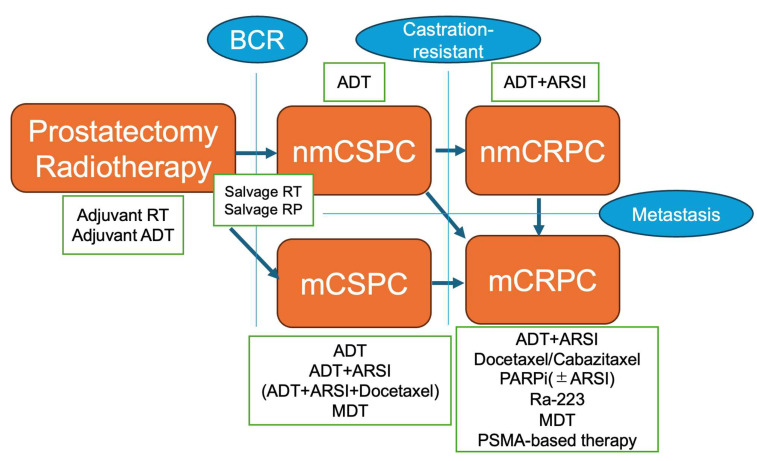
Proposed overview for treatment of prostate cancer from definitive therapy to metastatic castration-resistant prostate cancer. Abbreviations: BCR, biochemical recurrence; ADT, androgen deprivation therapy; ARSI, androgen receptor signaling inhibitor; nmCSPC, non-metastatic castration-sensitive prostate cancer; nmCRPC, non-metastatic castration-resistant prostate cancer; RT, radiotherapy; RP, radical prostatectomy; mCSPC, metastatic castration-sensitive prostate cancer; mCRPC, metastatic castration-resistant prostate cancer; MDT, metastasis direct therapy; PARPi, poly ADP-ribose polymerase inhibitor; Ra-223, radium-223; PSMA, prostate-specific membrane antigen.

The current treatment options for patients with PCa who have undergone definitive therapy after progression are shown in Figure 2. However, there have been no studies with strong evidence of the significance of definitive therapy for patients with mCRPC, and prospective studies are needed to verify its true benefits.

## 6. Conclusions

This review discusses the evidence for the oncologic outcomes of PCa after definitive therapy. Definitive therapy for PCa may improve the oncological outcomes; furthermore, RP may contribute to longer survival than RT. Therefore, definitive therapy for PCa should be performed for LPC and LAPC, if possible, to improve the oncological outcomes.

## Figures and Tables

**Table 1:** Comparison of surgical outcomes between ORP and RARP.

Study	Year	Number of Cases	Median Age, Years	≥Clinical T2 (%)	Median Operation Time, min	Median EBL, mL	Blood Transfusion Rate (%)
Krambeck et al. [37]	2008	ORP, 588; RARP, 294	ORP, 61.5; RARP, 60.5	ORP, 28.3; RARP, 27.2	ORP, 204; RARP, 236	NA	ORP, 13; RARP, 5.1
Rocco et al. [38]	2009	ORP, 240; RARP, 120	ORP, 63; RARP, 63	ORP, 39; RARP, 31	ORP, 160; RARP, 215	ORP, 800; RARP, 200	NA
Doumerc et al. [39]	2010	ORP, 502; RARP, 212	ORP, 60.1; RARP, 61.3	ORP, 58; RARP, 50	ORP, 148; RARP, 192	ORP <499 69.7 500–999 29.1 >1000 1.2 RARP <499 98.4 500–999 1.6 >1000 0	ORP, 2.0; RARP, 0.9
Kordan et al. [40]	2010	ORP, 414; RARP, 830	ORP, 61.5; RARP, 60.5	ORP, 31.2; RARP, 24.8	NA	ORP, 450; RARP, 100	ORP, 3.4; RARP, 0.8

Abbreviations: EBL, estimated blood loss; ORP, open radical prostatectomy; RARP, robot-assisted radical prostatectomy; NA, not applicable.

**Table 2:** Positive rates of surgical margins and oncological outcomes in patients with PCa who underwent RARP.

Study	PSM Rate	Number of PSMs (%)	Location of PSM (%)	BCR, Number (%)	Median Follow-up Period (Months)	Increased Risk of BCR
Komori et al. [54]	21.9%	Not applicable	Left: 9.6; right: 10.0; bilateral: 3.2; apex: 9.6; non-apical: 9.6	120 (14.7)	27.8	HR, 3.22; *p* < 0.001
Morizane et al. [55]	17.0%	Unifocal: 47.3; multifocal: 52.7	Apex: 54.3; mid-gland: 25.7; base: 25.7	61 (14.0)	52.4	HR, 3.281; *p* = 0.022
Yang et al. [56]	30.1%	Unifocal: 74.6; multifocal: 23.0	Apex: 27.7; mid-gland: 13.5; base: 73.8	97 (24.6)	29.3	HR, 1.725; *p* = 0.027
Porcaro et al. [57]	26.3%	Unifocal: 69.3; multifocal: 30.7	Not applicable	40 (8.7)	26	HR, 3.771; *p* < 0.001
JO et al. [58]	14.5%	Unifocal: 42.5; multifocal: 57.5	Apex: 43.7%; other site: 56.3%	152 (18.7)	Not applicable	HR, 3.123; *p* < 0.001

Abbreviations: PCa, prostate cancer; PSM, positive surgical margin; BCR, biochemical recurrence; HR, hazard ratio.

**Table 3:** Radiation therapy options for PCa management by risk category according to NCCN, AUA, and ASTRO guidelines.

PCa Risk and Metastatic Patterns	Conventional Fractionation EBRT	Moderate Hypofraction EBRT	Ultra Hypofraction EBRT	BT Alone	Combination Therapy of EBRT and BT Boost
Low-risk	◯	◯	◯	◯	
Favorable-intermediate risk	◯	◯	◯	◯	
Unfavorable-intermediate risk	◯	◯	◯	◯	◯
High-risk	◯	◯	◯		◯
Pelvic lymph node recurrence	◯	◯	◯		
Salvage therapy after surgery	◯	◯			
De novo oligometastasis		◯			

Abbreviations: PCa, prostate cancer; NCCN, National Comprehensive Cancer Network; AUA, American Urological Association; ASTRO, American Society for Radiation Oncology; EBRT, external-beam radiation therapy; BT, brachytherapy. ◯: recommendation.

**Table 4:** Oncological outcomes in patients with PCa who underwent definitive therapies.

Study	Treatment, Number	Median Follow-up Period	PCSM or PCSS		MFS		OS or OM	
			%	Statistical Analysis	%	Statistical Analysis	%	Statistical Analysis
Suárez et al. [9]	RP, 192; BT, 317; EBRT, 195	10 y	10-year PCSM: RP, 0%; BT, 1.9%; EBRT, 6.2%	HR (ref. RP): BT, 4.41 (95% CI, 0.69–28.29; *p* = 0.120); EBRT, 9.37 (95% CI, 1.53–57.21; *p* = 0.015)	NA	NA	10-year OS: RP, 85.3%; BT, 78.1%; EBRT, 73.3%	HR (ref. RP): BT, 1.36; (95% CI, 0.77–2.42; *p* = 0.292); EBRT, 1.40 (95% CI, 0.78–2.51; *p* = 0.222)
Li et al. [89]	LRP, 64; RT, 154	LRP, 53.5M; RT, 64 M	5-year PCSS: LRP, 93.3%; RT, 64.7%	*p* = 0.022	5-year: LRP, 48.0%; RT, 40.2%	*p* = 0.045	5-year OS: LRP, 93.3%; RT, 59.3%	*p* = 0.004
Hoffman et al. [90]	RP, 402; EBRT, 217	73 M	5-year PCSS: RP, 99.5%; EBRT, 99.0%	*p* = 0.10	NA	NA	5-year OS: RP, 97.7%; EBRT, 91.8%	*p* < 0.001
Caño-Velasco et al. [91]	RP, 145; EBRT, 141	RP, 152 M; EBRT, 97 M	5-year PCSS: RP, 95.7%; EBRT, 97.0%	*p* = 0.44	NA	NA	5-year OS: RP, 92.4%; EBRT, 89.2%	HR (ref. EBRT): RP, 0.48 (95% CI, 0.48–1.50; *p* = 0.04)
Koo et al. [92]	RP, 339; EBRT, 339	RP, 69M; EBRT, 60.5M	5-year PCSS: RP, 98.8%; EBRT, 99.5%	*p* = 0.576	5-year: RP, 33.3%; EBRT 41.7%	*p* = 0.778	5-year OS: RP, 94.7%; EBRT, 92.0%	*p* = 0.105
Aas et al. [93]	RP, 104; EBRT, 294	10y	10-year PCSM: RP, 1.5%; EBRT, 6.2%	HR (ref. RP): EBRT, 2.0 (95% CI, 1.03–3.69; *p* = 0.034)	NA	NA	10-year OM: RP, 9.3%; EBRT, 20.5%	NA

Abbreviations: PCa, prostate cancer; PCSM, prostate cancer-specific mortality; PCSS, prostate cancer-specific survival; MFS, metastatic-free survival; OS, overall survival; OM, overall mortality; RP, radical prostatectomy; BT, brachytherapy; EBRT, external-beam radiation therapy; HR, hazard ratio; CI, confidence interval; NA, not applicable; y, year; M, months.

## Data Availability

The data presented in this study are available upon request from the corresponding author. The data are not publicly available due to privacy and ethical reasons.
